# Can Serum Glypican-3 Be a Biomarker for Effective Diagnosis of Hepatocellular Carcinoma? A Meta-Analysis of the Literature

**DOI:** 10.1155/2014/127831

**Published:** 2014-10-14

**Authors:** Sheng-Li Yang, Xiefan Fang, Zao-Zao Huang, Xiang-Jie Liu, Zhi-Fan Xiong, Ping Liu, Hong-Yi Yao, Chang-Hai Li

**Affiliations:** ^1^Department of General Surgery, Liyuan Hospital, Tongji Medical College, Huazhong University of Science and Technology, Wuhan, Hubei 430077, China; ^2^Department of Pediatrics, College of Medicine, University of Florida, Gainesville, FL 32610, USA; ^3^Department of Rehabilitation, Liyuan Hospital, Tongji Medical College, Huazhong University of Science and Technology, Wuhan 430077, China; ^4^Department of Medicine and Division of Digestion Disease, Liyuan Hospital, Tongji Medical College, Huazhong University of Science and Technology, Wuhan, Hubei 430077, China; ^5^Hepatic Surgery Center, Tongji Hospital, Tongji Medical College, Huazhong Science and Technology University, Wuhan, Hubei 430030, China

## Abstract

*Objective*. This review is to evaluate the diagnostic value of serum GPC3 for hepatocellular carcinoma (HCC) due to conflicting results reported. *Methods*. NCBI PubMed and Embase were comprehensively searched for studies that have used serum GPC3 level as a diagnostic index for HCC. The quality of the included studies was assessed. Subgroup analyses were conducted to evaluate the sensitivity and specificity of GPC3 as a HCC marker. Statistical analysis was performed with the software STATA version 12.0. *Results*. A total of 22 studies were included. The qualities of included studies were relatively poor. Among them, 18 studies have shown that serum GPC3 is a specific biomarker for HCC, and the pooled sensitivity and specificity of these studies were 69 and 93%, respectively. The other 4 studies have reported conflicting results, which were not caused by races, infection status of HBV and HCV, or assay reagents but due to one common experimental design of enrolling liver cirrhosis patients as control subjects. *Conclusions*. This meta-analysis indicates that serum GPC3 is elevated in HCC patients compared with healthy individuals, but more studies are needed to evaluate its effectiveness to differentially diagnose HCC and liver cirrhosis.

## 1. Introduction

Primary hepatocellular carcinoma (HCC) is one of the most malignant tumors and is the most common cause of cancer-related death worldwide [1]. Nowadays, laparoscopic surgery and drug intervention with Sorafenib are widely utilized to treat HCC patients. But their 5-year survival rate remains low [2, 3] because many patients are diagnosed at the late stage of HCC and lose the opportunity of effective medical interventions. Thus, diagnosis of HCC at an early stage is of utmost importance for reducing HCC-associated mortality.

European Association for the Study of the Liver (EASL) recommends patients with liver diseases to do liver ultrasound and examine serum *α*-fetoprotein (AFP) level every six months [4]. However, ultrasound is an indirect diagnostic method with accuracy greatly depending on the skill of operators and has limited ability to distinguish HCC from nonneoplastic nodules [4]. Similarly, serum AFP level is not an accurate biomarker for HCC because its sensitivity only ranges from 40 to 65% [5]. Therefore, a novel biomarker with superior diagnostic accuracy is greatly desired.

Glypican-3 (GPC3) belongs to the glypican family of heparan-sulfate proteoglycans [6]. GPC3 is normally expressed in fetal liver and placenta and has negligible expression in normal adult liver [6]. Currently, many studies have found that GPC3 expression is increased in HCC tissues [7–9], even though its expression is absent in the hepatocytes of healthy individuals and hepatitis patients. Furthermore, serum GPC3 level is higher in HCC patients than that in healthy individuals and hepatitis patients. Thus, it has been suggested that serum GPC3 is a specific biomarker for HCC [10–27]. But other studies reported conflicting results [28–31]. In the present study, we performed a meta-analysis on the data from all published studies that have evaluated the diagnostic potential of serum GPC3 for HCC and concluded that serum GPC3 is a clinically relevant HCC biomarker.

## 2. Methods

### 2.1. Literature Search Strategy

We performed a comprehensive search on the peer-reviewed scientific literatures that are written in English and were published before May 20, 2014, in NCBI PubMed or EMBASE. The following search terms were used: (1) GPC3: glypican-3 and GPC3; and (2) HCC: HCC, hepatocellular carcinoma, liver cancer, liver cell carcinoma, and hepatic cell carcinoma. No restrictions on study design, year of publication, or publication type were set during initial database search. To avoid exclusion of relevant studies, we did not use keywords or indexing terms for diagnostic test accuracy. After filtering the studies based on the criteria listed in the next paragraph, we manually searched the reference lists of selected articles to identify more relevant publications.

### 2.2. Criteria for Inclusion and Exclusion of Published Studies

The inclusion criteria for articles were as follows: (1) studies investigated the diagnostic accuracy of serum GPC3 for HCC; (2) studies have reported calculable data on sample sizes of HCC and non-HCC patients, true positive (TP), true negative (TN), false positive (FP), and false negative (FN) values; and (3) article is written in English. The exclusion criteria were as follows: (1) studies conducted on animals; (2) studies that evaluated mRNA expression or DNA polymorphisms of GPC3 and did not provide the sensitivity or specificity of using GPC3 as a HCC marker; (3) letters, editorials, expert opinions, and reviews without original clinical data; (4) case reports and studies lacking control groups; and (5) duplicate reports.

### 2.3. Identification of Eligible Studies and Data Extraction

Initial screening for potentially eligible studies was carried out by author Sheng-Li Yang based on the titles and abstracts of articles. Two authors Sheng-Li Yang and Xiefan Fang independently reviewed and included eligible studies based on the criteria described above. Disagreements were resolved by discussion or consulting with author Zao-Zao Huang. After all the eligible studies were identified, the following characteristics were retrieved from each study: authors, geographic distribution of patients, study design, number of patients, reference test, methods of measurement, cutoff values, and raw data including TP, FP, TN, and FN results.

### 2.4. Quality Assessment of Included Studies

Authors Sheng-Li Yang and Xiefan Fang independently assessed qualities of the eligible studies using the recommended checklist of Quality Assessment of studies of Diagnostic Accuracy included in Systematic reviews (QUADAS, Cochrane Collaboration). Each of the eleven items in the QUADAS checklist was scored as “yes,” “no,” or “unclear” [13]. Any disagreements in quality assessments were resolved by discussion or consulting with author Zao-Zao Huang.

### 2.5. Data Analysis

For each study, we calculated sensitivity, specificity, positive likelihood ratios (LR+), and negative likelihood ratios (LR−), as well as their corresponding 95% confidence intervals (95% CIs). The data were visualized as forest plots and receiver operating characteristic curves (ROC). Heterogeneity of the retrieved data from eligible studies was evaluated by using the Q statistics, with a significance level at *P* < 0.10. The *I*-square value, a quantitative measurement of inconsistency across different studies [32], was also calculated. *I*-square value typically ranges from 0 (no observed heterogeneity) to 100% (maximal heterogeneity), and an *I*-square value ≥ 50% is considered to represent substantial heterogeneity. If heterogeneity across studies was not identified, the fixed-effects model was used for meta-analysis. Otherwise, the random-effects model (DerSimonian-Laird method) was used [33]. Publication bias was measured by Deeks' funnel plot asymmetry test [34]. The meta-analysis was performed by the statistical software STATA version 12.0 (StataCorp LP, College Station, TX, USA), and statistical significance was defined as *P* value less than 0.05.

### 2.6. Subgroup Analysis

Because substantial heterogeneity existed in the included studies, we performed subgroup meta-analysis by dividing studies into one group that found that GPC3 has diagnostic value in HCC detection and another group that claimed that GPC3 has no diagnostic value for HCC. The Spearman approach was applied to test if the heterogeneity can be explained by a threshold effect.

## 3. Results

### 3.1. Study Selection

**Figure 1:**
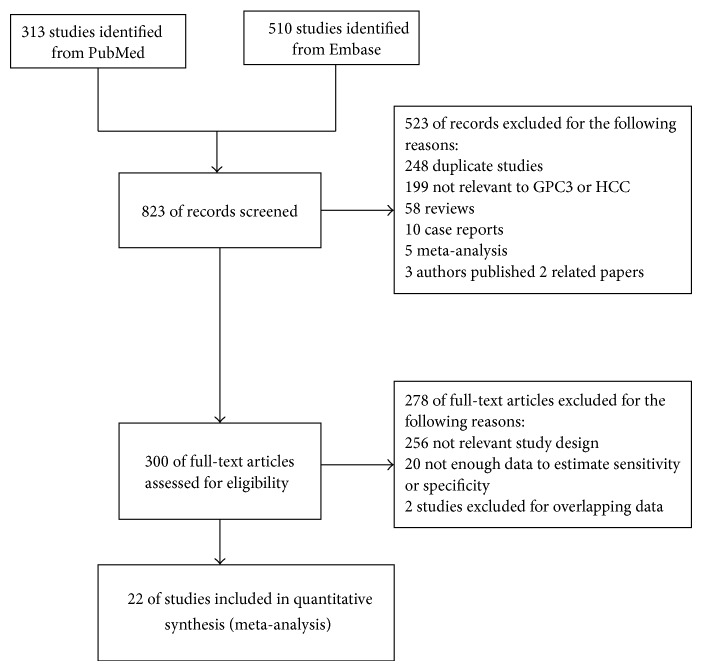
Study selection process.

A total of 823 potentially relevant articles were identified by searches in NCBI PubMed and Embase. After reviewing their titles and abstracts, 523 articles, including duplicate studies, case reports, reviews, and comments, were excluded. After reviewing the full texts, 256, 20, and 2 studies were excluded due to irrelevant study design, insufficient data to estimate sensitivity or specificity, and publishing overlapping data, respectively. In the references of the retrieved studies, no additional articles met our inclusion criteria. Finally, twenty-two studies were included for meta-analysis [10–31] (Figure 1). The characteristics of the included studies are shown in Table 1. The eight studies from China mostly enrolled hepatitis B virus- (HBV-) associated HCC patients [10, 13, 14, 17, 18, 20, 22, 28]. The five studies from Egypt mainly had hepatitis C virus- (HCV-) associated HCC patients [12, 15, 16, 19, 21]. The study from the United Kingdom had HCC patients without virus infection [24]. There were differences in what human subjects were used as experimental controls among the included studies. Some studies enrolled healthy individuals as controls [13], some enrolled patients with hepatitis or liver cirrhosis [11, 12, 16, 17, 22–27, 29–31], and others combined both healthy people and patients with hepatitis or liver cirrhosis as one control group [10, 14, 15, 18–21, 28].

### 3.2. Quality of the Studies

**Figure 2:**
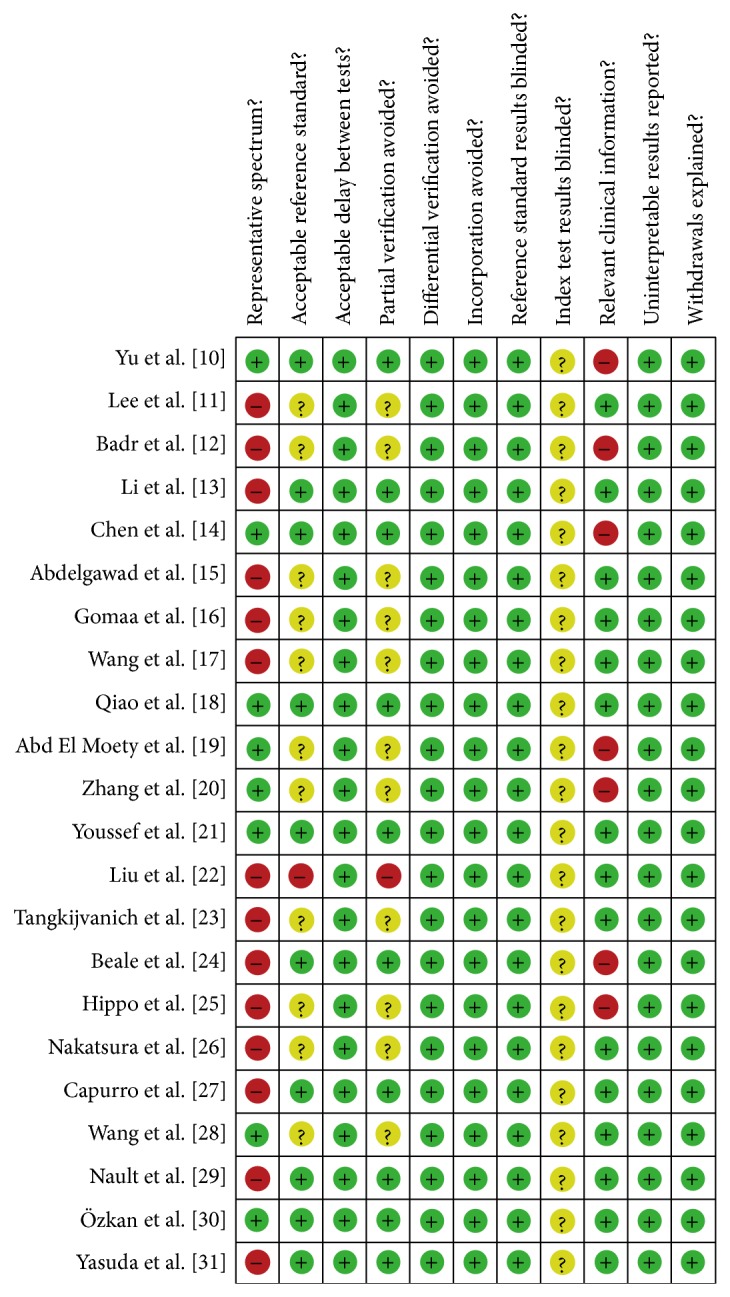
Summary of methodological quality of included studies on the basis of review authors' judgments on the 11 items of QUADAS checklist for each study.

The results of QUADAS quality assessment of the included studies are shown in Figure 2. The quality was not satisfactory. The major biases of the included studies fell in the domains of “representative spectrum,” “index test results blinded,” and “relevant clinical information.” In the domain of “representative spectrum,” only eight studies enrolled both healthy people and patients with hepatitis or liver cirrhosis as controls [10, 14, 15, 18–21, 28]. In the domain of “index test results blinded,” all studies reported the diagnostic standard for HCC; however, no studies have stated whether the index results were blindly interpreted or not. In the domain of “relevant clinical information,” seven studies did not provide enough relevant clinical information of the HCC patients [10, 12, 14, 19, 20, 24, 25].

### 3.3. Diagnostic Accuracy of GPC3 for HCC

**Figure 3:**
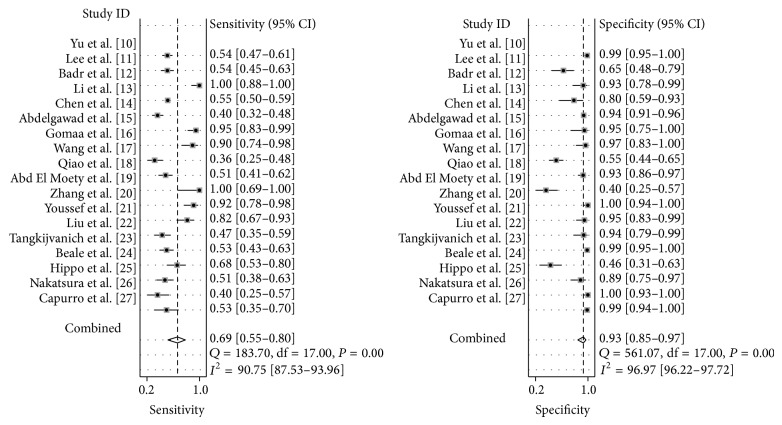
Forest plots of sensitivity and specificity of using GPC3 as a diagnostic marker for hepatocellular cancer (HCC) in the eighteen studies included for meta-analysis [10–27].

**Figure 4:**
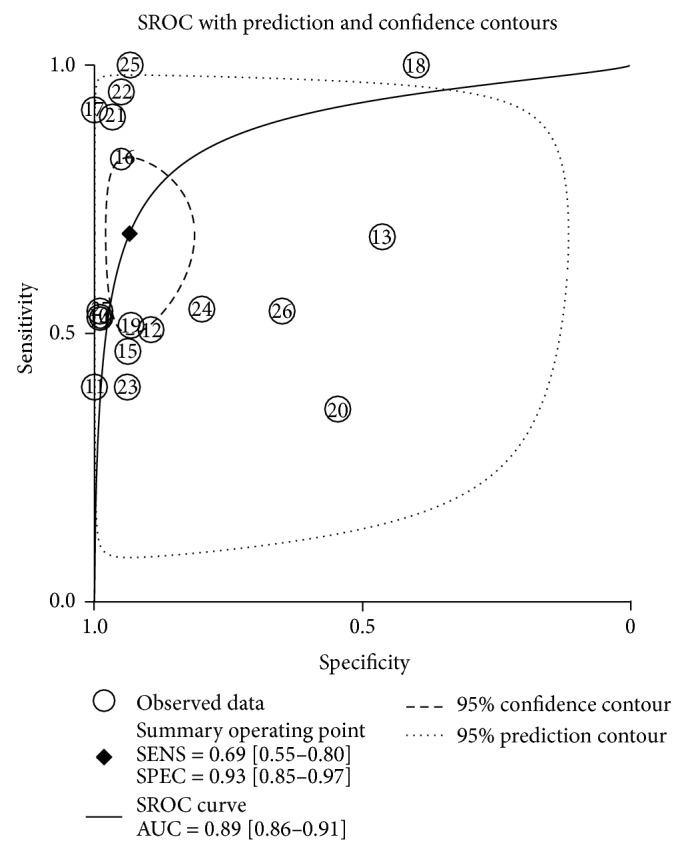
Summary receiver operating characteristic curves (SROC) from the hierarchical summary receiver operating characteristic model generated from the eighteen studies that found that GPC3 is a diagnostic marker for HCC [10–27].

Among the twenty-two studies, eighteen of them have demonstrated that serum GPC3 level is higher in HCC patients than that in control subjects, which include healthy individuals and patients with hepatitis or liver cirrhosis [10–27] (Table 1), whereas four studies have claimed that GPC3 is not a diagnostic marker for HCC because serum GPC3 level is lower in HCC patients than that in patients with liver cirrhosis [28–31]. These four studies did not provide TP, TN, FP, or FN values [28–31] (Table 2). Thus, we divided the studies into two subgroups, and only the subgroup that provided the values of TP, TN, FP, and FN was used for meta-analysis [10–27]. The forest plots of sensitivity (TP rate) and specificity (FP rate) for the 18 studies are shown in Figure 3. The sensitivity and specificity of the studies were plotted in a hierarchical summary receiver operating characteristic graph (SROC, Figure 4). The sensitivity of using GPC3 for HCC diagnosis ranged from 36 to 100%, and the specificity ranged from 42 to 100%. The average sensitivity and specificity were 69% (95% CI was 55–80%) and 93% (95% CI was 85–97%), respectively. The overall diagnostic odds ratio (DOR) and the area under SROC were 31 (95% CI was 11–92) and 0.89 (95% CI was 0.86–0.91), respectively. *I*-square values of sensitivity and specificity were 90.71 and 97.30, respectively, indicating that substantial heterogeneity existed among the eligible studies. The Spearman correlation coefficient between the logic of sensitivity and the logic of 1-specificity was 0.043 (*P* = 0.86), indicating that the heterogeneity among eligible studies was not caused by the threshold effect.

### 3.4. Publication Bias

**Figure 5:**
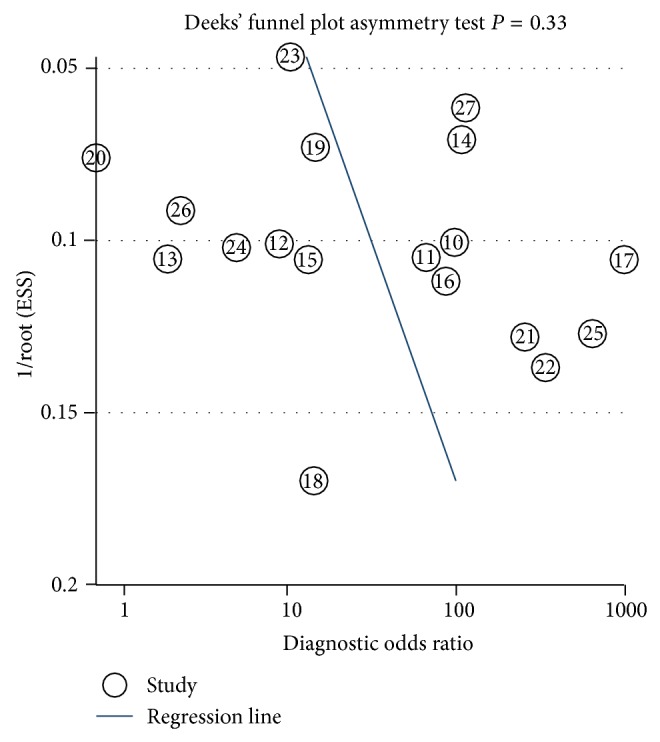
Linear regression test of funnel plot asymmetry. The statistically nonsignificant *P* value of the slop coefficient indicates symmetry of the data and a low likelihood of publication bias.

We used Deeks' funnel plot asymmetry test to evaluate publication bias among the included studies. The slope coefficient of the regression line had a *P* value of 0.33 indicating that the data were symmetric and did not have a likelihood of publication bias (Figure 5).

## 4. Discussion

HCC is one of the most lethal malignancies with a survival rate less than 10% and its incidence is increasing worldwide [35]. Specific and sensitive methods are urgently needed to accurately diagnose HCC at an early stage. Detection of serum biomarkers is a simple, rapid, and noninvasive method to diagnose HCC. Currently, several serum biomarkers for HCC, including AFP, AFP-L3, Golgi protein 73 (GP73), and GPC3 have been identified [36]. GPC3 is a 60 kDa cell-surface protein that belongs to the heparan-sulfate proteoglycan family, which contains GPC1, 2, 3, 4, 5, and 6. GPC3 is cleaved by furin between Arg358 and Ser359 to release a 40 kDa protein with the amino (N) terminal and a 30 kDa protein with the membrane-bound carboxyl (C) terminal [37]. The protein derived from the N-terminal of GPC3, also known as soluble GPC3 (sGPC3), is detected in the sera of HCC patients and is a potential serum marker for HCC diagnosis.

In this meta-analysis, we identified twenty-two studies that have investigated the diagnostic accuracy of serum GPC3 for HCC. Eighteen of them demonstrated that GPC3 is an ideal HCC diagnostic marker with pooled sensitivity, specificity, LR+, and LR− of 69%, 94%, 10.50, and 0.34, respectively [10–27]. They have found that serum GPC3 is elevated in HCC patients compared with healthy individuals and patients with hepatitis or liver cirrhosis [10–27]. But four studies have found lowered or equivalent serum levels of GPC3 in HCC patients compared with liver cirrhosis patients [28–31]. With careful comparison, we found that HBV or HCV infection and source of GPC3 antibodies were not the culprits for this discrepancy. Instead, the possible reason is that GPC3 is not able to differentially diagnose HCC and liver cirrhosis, and thus the studies that used liver cirrhosis patients as controls have found conflicting results [28–31].

We found that the infection status of HBV or HCV in HCC and control subjects were different in the included studies. In the studies from Egypt and Japan, more than 60% of the enrolled patients with HCC were infected with HCV, and approximately 15–20% of patients were infected with HBV [12, 15, 16, 31]. In contrast, approximately 85 and 10% of Chinese HCC cases were associated with HBV and HCV infection, respectively [13, 17, 22, 28, 38]. The four studies which held the controversial views were not conducted in the same region, but in France [29], China [28], Japan [31], and Turkey [30]. Therefore, the virus associated with HCC has no apparent influences on the diagnostic accuracy of GPC3.

The assay reagents used to measure serum GPC3 levels differed among the included studies. Some studies used self-made antibodies. For example, Hippo et al. used an antibody that binds to the N-terminal portion of GPC3 cleaved at Arg358 (amino acids 25–358) [25]. Capurro et al. used the anti-GPC3 monoclonal antibody “clone 1G12” that recognizes the last 70 amino acids of the C-terminal of the core protein (amino acids 491–560) [27]. The other studies used commercially available kits from different manufacturers, and their antibodies may target different regions of GPC3 [11, 12, 16–18]. The four conflicting studies used kits from Wuhan Cusabio Biotech, Usabio Biotech, Wuhan EIAab, and BioMosaics [28–31], which were also used in the other studies that found that GPC3 is a useful HCC biomarker [11, 13, 16, 19, 22, 24]. Therefore, the source of antibodies is not the reason that led to inconsistent results among the included studies.

We noticed that one common experimental design of the four conflicting studies is that they enrolled liver cirrhosis patients as control subjects, even though they had various complications, including alcoholic cirrhosis and HBV- or HCV-associated cirrhosis [28–31]. In fact, all four studies observed that serum GPC3 level is higher in liver cirrhosis patients than that in HCC patients [28–31]. Therefore, elevated serum GPC3 level is not a unique feature in HCC and is also seen in liver cirrhosis patients. But because measuring serum GPC3 level is a quick assay, it still could be the first step to screen for HCC. If elevated GPC3 level is detected, further testing should be done to confirm HCC diagnosis.

Considering that the included studies have substantial heterogeneity and part of them held the opposite views, a well-designed prospective study with larger cohorts should be performed to rigorously evaluate the diagnostic accuracy of GPC3 and determine if it has a better diagnostic value compared with other known HCC serum biomarkers. In addition, experimental design should be improved in the following areas: (1) double-blind studies should be designed to avoid bias; (2) the cohorts of healthy individuals, hepatitis patients, and liver cirrhosis patients should be compared as separate groups; (3) the study could use two or more different GPC3 antibodies to measure GPC3 level; (4) it is important to examine the stability of GPC3 during long-term storage. Half of the included studies measured GPC3 level in frozen serum, but it is not sure if GPC3 would be degraded after long-term storage. If the stability of serum GPC3 in long-term storage is not good, the diagnostic performance of serum GPC3 may be greatly affected.

To our knowledge, several studies have performed comprehensive reviews on using serum GPC3 as a diagnostic indicator for HCC [39–41]. However, Huang et al. only provided a protocol for meta-analysis and did not make a conclusion in his report [39]. Xu et al. performed a meta-analysis on merely ten studies and agreed upon that GPC3 is a good diagnostic marker for HCC, and they did not include the conflicting studies that we have found [41]. Liu et al. included twelve studies for their analysis and found inconsistent results among literatures as we did [40]. They suggested that GPC3 has moderate diagnostic accuracy for HCC and additional studies with larger sample size should be done to make a conclusion [40]. Compared with the previous reviews, our meta-analysis included more patients (5931) and studies (22), including those published after 2013. More importantly, we identified that having liver cirrhosis patients as control subjects is the potential cause for the inconsistent results in literatures, whereas race, country, infection with HBV or HCV, or assay reagents are not responsible for the discrepancies. We found that elevated GPC3 level is not unique for HCC but is also found in liver cirrhosis patients. These findings are valuable for future experimental designs to further explore the link between GPC3 and HCC.

## 5. Conclusion

In summary, our meta-analysis indicates that serum GPC3 level is elevated in HCC patients compared with healthy individuals. But whether GPC3 is an index to differentially diagnose HCC and liver cirrhosis is still uncertain.

## Figures and Tables

**Table 1:** Characteristic and methodology assessment of the included studies.

First author, year, country	Characteristics of HCC	Characteristics of controls	GPC3				
			Assay type	Cut-off value	Antibody for detection	HCC value (ng/mL)	Controls value (ng/mL)
Yu [10], 2014, China	NA	LC or hepatitis patients; healthy individuals	Chemiluminescent immunoassay	30 ng/mL	GPC3 8G6 mcAb and 7D11 mcAb (Millipore Corporation)	108.67 ± 230.04	3.99 ± 7.68

Lee [11], 2014, Korea	62.5% were HBV associated	CLD (HCV) patients; 50% had liver cirrhosis	ELISA	73 ng/mL	ELISA kit (Wuhan Cusabio Biotech)	75.8 ± 117.5	66.4 ± 33.2

Badr [12], 2014, Egypt	NA	LC (HCV) patients	ELISA	240 ng/mL	ELISA kit (Wuhan Uscn)	551.47 ± 185.25	98.23 ± 73.54

Li [13], 2013, China	HBV associated HCC	Healthy individuals	ELISA	NA	ELISA kit (usabio Biotech)	12.63 ± 2.93 for patients with AFP <400 *µ*g/L; 20.20 ± 5.41 for patients with AFP ≥ 400 *µ*g/L	1.92 ± 0.95

Chen [14], 2013, China	NA	LC or hepatitis patients; healthy individuals	ELISA	25.25 ng/mL	7C8 and GP9 mcAb (self-made)	99.94 ± 267.2	19.44 ± 50.88 for LC patients; 10.45 ± 46.02 for chronic hepatitis; 4.14 ± 31.65 for healthy controls

Abdelgawad [15], 2013, Egypt	67.5% were HCV associated; 17.5% were HBV associated	LC patients; healthy individuals	ELISA	4.9 ng/mL	ELISA kit (Wuhan Uscn)	7.7	3.24

Gomaa [16], 2012, Egypt	12.9% were HBV associated; 87.1% were HCV associated	LC patients with HCV/HBV	ELISA	5.41 ng/mL	ELISA kit (BioMosaics)	8.13 ± 3.25	3.14 ± 1.16

Wang [17], 2012, China	HBV associated	LC patients with HBV	ELISA	NA	ELISA kit (Wuhan Huamei)	NA	NA

Qiao [18], 2011, China	76.2% were HBV associated; 7.9% were HCV associated	LC (HBV/HCV) or hepatitis patients; healthy individuals	ELISA	26.8 ng/mL	ELISA kit (USCN life science)	29.29 ± 17.34	12.09 ± 9.69 for LC patients; 9.98 ± 9.60 for chronic hepatitis patients; 5.93 ± 5.46 for healthy controls

Abd El Moety [19], 2011, Egypt	HCV associated	LC or hepatitis patients; healthy individuals	ELISA	2.0 ng/mL	ELISA kit (BioMosaics)	34.63 ± 23.8	NA

Zhang [20], 2010, China	NA	LC or hepatitis patients with HCV/HBV; healthy individuals	Immunoassay	3.10 ng/mL	Self-made	116.8 ± 98.6	24.60 ± 24.01 for LC patients; 6.73 ± 12.2 for hepatitis B patients; 13.67 ± 15.68 for hepatitis C patients; 0.86 ± 1.12 for healthy controls

Youssef [21], 2010, Egypt	HCV and HBV associated	LC patients with HBV/HCV; healthy individuals	ELISA	4.6 ng/mL	ELISA kit (BioMosaics)	NA	NA

Liu [22], 2010, China	84% were HBV associated; 16% were HCV associated	LC patients with HBV/HCV	ELISA	300 ng/L	ELISA kit (BioMosaics)	NA	NA

Tangkijvanich [23], 2010, Thailand	59% were HBV associated; 11% were HCV associated	LC or hepatitis patients with HBV/HCV	ELISA	NA	Self-made	46.3 (0–7826.6)^■^	0 (0–43.6)^■^

Beale [24], 2008, UK	60% had ALD and 40% patients had NAFLD	LC patients with ALD/NAFLD	ELISA	NA	ELISA kit (BioMosaics)	161.41 ± 422.33	125.41 ± 281.05

Yoshitaka Hippo [25], 2004, Japan	NA	LC patients	ELISA	2.0 ng/mL	Self-made	4.84 ± 8.91	1.09 ± 0.74

Nakatsura [26], 2003, Japan	12.1% were HBV associated; 40.9% were HCV associated	LC patients with HBV/HCV/PBC/AIH	ELISA	10 U/mL	Self-made	NA	NA

Capurro [27], 2003, Canada	44.1% were HBV associated; 27.2% were HCV associated; 14.7% had ALD	LC or hepatitis patients with HBV/HCV	ELISA	117 ng/mL	Self-made 1G12 antibody	NA	NA

Wang [28], 2014, China	84.5% were HBV associated; 9.5% were HBV associated; 3.5% were alcoholic cirrhosis associated	LC or hepatitis (HBV) patients; healthy individuals	ELISA	NA∗	ELISA kit (usabio Biotech)	4.55 ± 3.5	9.03 ± 4.1 for LC patients; 4.56 ± 3.2 for HBV chronic hepatitis patients; 6.71 ± 1.8 for healthy controls

Nault [29], 2013, France	With alcoholic cirrhotic, 46.4% were early stage	Alcoholic cirrhotic patients	ELISA	NA∗	ELISA kit (Wuhan Cusabio Biotech)	1.4 ± 0.5 (early stage) 4.2 ± 1.8 (advanced stage)	2.5 ± 1.4

Özkan [30], 2011, Turkey	52% were HBV associated; 23% were HCV associated	LC patients with HBV/HCV/HDV/AIH/Wilson disease/Cryptogenic liver disease; healthy individuals	ELISA	NA∗	ELISA kit (Wuhan EIAab)	5.13 ± 22.7	5.51 ± 59.2

Yasuda [31], 2010, Japan	16% were HBV associated; 77.5% were HCV associated	CLD patients with HBV/HCV	ELISA	NA∗	ELISA kit (BioMosaics)	924.8 (495.2, 1335.6)^▲^	1161.6 (762.0, 1784.0)^▲^

NA: data are not available; HBV: hepatitis B virus; HCV: hepatitis C virus; LC: liver cirrhosis; CLD: chronic liver disease; ALD: alcoholic liver disease; ALC: alcoholic liver cirrhosis; NAFLD: nonalcoholic fatty liver diseases; PBC: primary biliary cirrhosis; AIH: autoimmune hepatitis; ^▲^mean median (25% and 75% quartiles); ^■^mean median (ranges); ∗cutoff values were not provided because these studies found serum GPC3 has no correlation with HCC.

**Table 2:** Patients enrolled in the selected studies used for meta-analysis.

Author (ref.)	Case	Control	TP	FP	FN	TN
Yu et al. [10]	192	101	104	1	88	100
Lee et al. [11]	120	40	65	14	55	26
Badr et al. [12]	30	30	30	2	0	28
Li et al. [13]	605	25	330	5	275	20
Chen et al. [14]	155	440	62	27	93	413
Abdelgawad et al. [15]	40	20	38	1	2	19
Gomaa et al. [16]	31	30	28	1	3	29
Wang et al. [17]	78	97	28	44	50	53
Qiao et al. [18]	101	88	52	6	49	82
Abd El Moety et al. [19]	10	40	10	24	0	16
Zhang et al. [20]	36	93	33	0	3	56
Youssef et al. [21]	40	40	33	2	7	38
Liu et al. [22]	75	32	35	2	40	30
Tangkijvanich et al. [23]	100	100	53	1	47	99
Beale et al. [24]	50	41	34	22	16	19
Hippo et al. [25]	69	38	35	4	34	34
Nakatsura et al. [26]	40	50	16	0	24	50
Capurro et al. [27]	34	91	18	1	16	90
Wang et al. [28]	84	173	NA	NA	NA	NA
Nault et al. [29]	125	170	NA	NA	NA	NA
Özkan et al. [30]	75	55	NA	NA	NA	NA
Yasuda et al. [31]	200	200	NA	NA	NA	NA

TP: true positive; FP: false positive; TN: true negative; FN: false negative; NA: data are not available.
